# Green-Synthesized Nanomaterials from Edible and Medicinal Mushrooms: A Sustainable Strategy Against Antimicrobial Resistance

**DOI:** 10.3390/pharmaceutics17111388

**Published:** 2025-10-27

**Authors:** Gréta Törős, Hassan El-Ramady, Duyen H. H. Nguyen, Walaa Alibrahem, Nihad Kharrat Helu, Reina Atieh, Arjun Muthu, Szintia Jevcsák, Dávid Semsey, Neama Abdalla, Tamer Elsakhawy, Alexandra Florence Tóth, Péter Tamás Nagy, József Prokisch

**Affiliations:** 1 Institute of Animal Science, Biotechnology and Nature Conservation, Faculty of Agricultural and Food Sciences and Environmental Management, University of Debrecen, Böszörményi Street 138, 4032 Debrecen, Hungary; nguyen.huu.huong.duyen@agr.unideb.hu (D.H.H.N.); semi@gmail.hu (D.S.); jprokisch@agr.unideb.hu (J.P.); 2 Doctoral School of Animal Husbandry, Faculty of Agricultural and Food Sciences and Environmental Management, University of Debrecen, Böszörményi Street 138, 4032 Debrecen, Hungary; 3 Soil and Water Department, Faculty of Agriculture, Kafrelsheikh University, Kafr El-Sheikh 33516, Egypt; hassan.elramady@agr.kfs.edu.eg; 4 Institute of Life Sciences, Vietnam Academy of Science and Technology, 9/621 Vo Nguyen Giap Street, Linh Trung Ward, Thu Duc City 700000, Ho Chi Minh, Vietnam; 5 Doctoral School of Nutrition and Food Science, University of Debrecen, Böszörményi Street 138, 4032 Debrecen, Hungary; atieh.reina@mailbox.unideb.hu (R.A.); arjun.muthu@agr.unideb.hu (A.M.); 6 Doctoral School of Health Sciences, University of Debrecen, Egyetem Tér 1, 4028 Debrecen, Hungary; walaaeb@mailbox.unideb.hu (W.A.);; 7 Institute of Agricultural Chemistry and Soil Science, Faculty of Agricultural and Food Sciences and Environmental Management, University of Debrecen, 138 Böszörményi Street, 4032 Debrecen, Hungary; 8 Institute of Food Technology, Faculty of Agricultural and Food Sciences and Environmental Management, University of Debrecen, 138 Böszörményi Street, 4032 Debrecen, Hungary; jevcsak@agr.unideb.hu; 9 Plant Biotechnology Department, Biotechnology Research Institute, National Research Centre, 33 El Buhouth St., Dokki, Giza 12622, Egypt; na.abdel-aal@nrc.sci.eg; 10 Microbiology Department, Soil, Water and Environment Research Institute, Sakha Agricultural Research Station, Agriculture Research Center, Kafr El-Sheikh 33717, Egypt; drelsakhawy@arc.sci.eg; 11 Faculty of Agricultural and Food Sciences and Environmental Management, Institute of Water and Environmental Management, University of Debrecen, Böszörményi Street 138, 4032 Debrecen, Hungary; toth.florence@agr.unideb.hu (A.F.T.); nagypt@agr.unideb.hu (P.T.N.)

**Keywords:** myco-nanoparticles, antimicrobial resistance, green synthesis, edible mushrooms, nanocarriers, drug delivery

## Abstract

Antimicrobial resistance (AMR) poses an escalating global health crisis, projected to cause up to 10 million deaths annually by 2050. Conventional antibiotics are increasingly ineffective due to microbial adaptation, overuse, and disruption of gut microbiota. Nanotechnology offers promising alternatives, but traditional nanoparticle synthesis often relies on toxic chemicals and energy-intensive processes. This review explores mushroom-derived nanoparticles (myco-NPs) as sustainable, eco-friendly antimicrobials. Edible and medicinal mushrooms contain bioactive compounds, including polysaccharides, flavonoids, and proteins, that act as reducing and stabilizing agents in nanoparticle biosynthesis. Myco-NPs exhibit antimicrobial activity through membrane disruption, oxidative stress, immune modulation, and biofilm inhibition, while also demonstrating synergistic effects with antibiotics and potential roles in regulating the gut microbiota. Recent advances highlight their potential applications in medicine, food safety, and environmental protection. However, challenges remain regarding standardization, safety evaluation, and large-scale production. We emphasize interdisciplinary collaboration as essential to translating mushroom-based nanotechnology into effective clinical and industrial solutions.

## 1. Introduction

Antimicrobial resistance (AMR) is accelerating at an alarming pace, threatening to reverse decades of medical progress. The World Health Organization warns that by 2050, AMR could lead to 10 million deaths annually, surpassing the mortality of cancer and placing immense strain on global healthcare systems [1,2]. The overuse and misuse of antibiotics, combined with microbial evolution and disrupted gut microbiota, have rendered many conventional drugs ineffective. This escalating crisis underscores the urgent need for alternative, sustainable antimicrobial strategies that surpass traditional antibiotics [3,4,5].

Nanotechnology has emerged as a promising solution to AMR, offering antimicrobial agents with unique mechanisms of action that include membrane disruption, oxidative stress via reactive oxygen species (ROS), and intracellular damage [6,7]. These multifaceted modes reduce the risk of resistance development.

However, conventional nanoparticle (NP) synthesis often relies on toxic solvents, high energy consumption, and generates environmentally hazardous waste, limiting their real-world applicability, particularly in food, health, and environmental contexts [8,9,10].

In response, green nanotechnology has gained traction by leveraging natural, renewable sources for the biosynthesis of nanoparticles [11,12]. Recent advancements have shown that biological sources such as edible and medicinal mushrooms contain bioactive compounds, flavonoids, polyphenols, and polysaccharides that act as reducing and stabilizing agents in NP formation [13]. These biosynthesized nanomaterials are not only functional and biodegradable but also exhibit antibacterial, antifungal, antihelminthic, antiviral, and prebiotic properties, making them valuable tools in fighting AMR and restoring gut microbial balance [2,14].

Building on this approach, edible and medicinal mushrooms offer a robust and underexplored platform for myco-nanotechnology. Fungi such as *Ganoderma lucidum*, *Cordyceps militaris*, and *Lentinula edodes* have long been utilized in traditional medicine and are recognized for their bioactive compounds, including phenolics, flavonoids, enzymes, and beta-glucans, that serve dual roles as bioreductants and antimicrobial agents [15,16,17]. Recent advancements in biosynthesis techniques are further elaborated in the production of silver nanoparticles from *Agaricus bisporus*. Their findings highlight the multifaceted applications of these nanoparticles, including their antibacterial, antibiofilm, and antioxidant activities. The review highlights the role of mycogenesis in producing eco-friendly nanomaterials, underscoring the notion that green synthesis not only offers an alternative to conventional methods but also aligns with biotechnological advancements aimed at ensuring food safety and public health [18].

The green synthesis of nanomaterials from mushrooms is not only aligned with the principles of sustainable chemistry but also taps into the therapeutic legacy and biological potency of these organisms [19]. In addition, mushroom-derived nanoparticles (myco-NPs) may influence gut microbiota composition, presenting new avenues in both infection control and host immune modulation [20].

The rapid emergence of multidrug-resistant strains compromises treatment efficacy and increases global mortality. These challenges reinforce the urgent need for alternative antimicrobial strategies, such as nanotechnology-based and biologically derived nanoparticles, that can circumvent traditional resistance pathways and restore therapeutic effectiveness [21].

This review critically evaluates mushroom-derived nanoparticles (myco-NPs) as eco-friendly antimicrobial agents, with particular emphasis on the relationships between their synthesis, structural characteristics, and biological activity. It further explores their translational potential as sustainable alternatives to conventional antimicrobials in the context of the global antimicrobial resistance (AMR) crisis. We aim to highlight the translational potential of myco-nanotechnology in addressing AMR while promoting circular bioeconomy and gut microbiota health.

## 2. Methodology of the Review

A systematic search was conducted using PubMed, ScienceDirect, SpringerLink, and Google Scholar for peer-reviewed articles published between 2019 and 2025. Keywords included “nanomaterials,” “green synthesis,” “mushrooms,” “myco-nanoparticles,” “antimicrobial activity,” and “gut microbiota.” Studies were included if they reported the synthesis, characterization, or biological activity of nanoparticles derived from edible or medicinal mushrooms. Exclusion criteria were lack of full text, limited methodological transparency, or conference abstracts only. Articles were evaluated for quality, author expertise, and relevance. Selected studies were thematically organized to highlight synthesis–structure–activity relationships and translational applications.

## 3. Bioactive Compounds in Mushrooms for NP Synthesis

**Figure 1:**
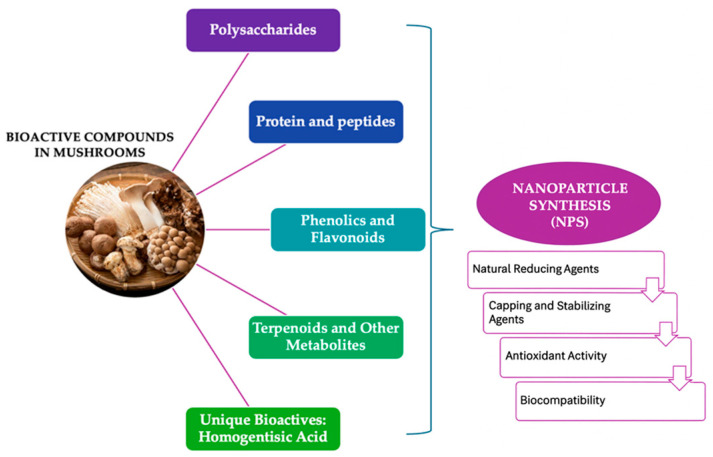
Bioactive Compounds in Mushrooms and Their Functional Roles in Nanoparticle Synthesis.

Mushrooms contain various bioactive compounds that serve as natural reducing, capping, and stabilizing agents, potentially benefiting the synthesis of nanoparticles (NPs). The bioactive compounds in mushrooms include polyols and carbonyl groups [22]. This mycosynthesis approach yields NPs with enhanced stability, biocompatibility, and biological activities, including antimicrobial and antioxidant properties [13,23]. The primary bioactive compound classes from different mushroom species are summarized. Figure 1. and Table 1 includes polysaccharides, proteins and peptides, phenolics and flavonoids, terpenoids, and unique metabolites like homogentisic acid, as primary contributors to NPs formation [24,25,26,27].

### 3.1. Polysaccharides: β-Glucans, Chitin, and Mannogalactans

Polysaccharides are the most studied class of mushroom bioactive compounds used for NPs synthesis due to their dual reducing and stabilizing capacities. Among the polysaccharides derived from mushrooms utilized in nanoparticle synthesis, β-glucans, chitin, and mannogalactans are the most extensively studied.

The polysaccharide extracts of mushroom species such as *Agaricus bisporus*, *Phellinus linteus*, *G. lucidum*, and *Pleurotus ostreatus* are used for silver nanoparticle (AgNPs) synthesis [27,28]. β-glucans, in particular, possess abundant hydroxyl groups that can donate electrons to reduce Ag^+^ ions to Ag^0^, while simultaneously forming a capping layer that stabilizes the NPs and prevents aggregation. This mechanism enables the production of controlled particle sizes, typically ranging from 10 to 70 nm, depending on the synthesis conditions [29]. Chitin and mannogalactans also contribute to NPs stabilization through their high molecular weight and functional group diversity, enhancing colloidal stability and biocompatibility [36].

### 3.2. Proteins and Peptides

Similarly to polysaccharides, proteins and peptides from mushroom extracts act as both reducing agents and capping molecules in the synthesis of NPs. For instance, *G. lucidum* mycelial filtrates and *Volvariella volvacea* extracts have successfully produced AgNPs and gold nanoparticles (AuNPs) by forming the amide linkages and amino or carboxylate groups on proteins, which reduce metal binding [24,25]. *A. bisporus* extract synthesized spherical high-stability AuNPs with an average size of 25 nm, demonstrating antifungal activity against *Aspergillus* species [22]. *Ganoderma* extract produced stable AgNPs with an average size of 2 nm [30]. Synthesized NPs from protein mushroom extracts often result in NPs with sizes ranging from 2 to 70 nm (Table 1). Fourier-transform infrared spectroscopy (FTIR) studies have confirmed that proteins from mushrooms can enhance the stability of NPs and prevent agglomeration [25].

### 3.3. Phenolics and Flavonoids

Phenolic acids, polyphenols, and flavonoids from mushroom extracts with antioxidant capacity, potentially enhanced the properties of synthesized NPs (Table 1). These compounds have been reported for the synthesis of AgNPs and ZnNPs, typically with sizes ranging from 10 to 200 nm [31,32]. The electron-donating capacity of phenolic hydroxyl groups enables metal ion reduction, while their aromatic structures cap NPs. For instance, methanolic extracts of *L. edodes*, rich in flavonoids and polyphenols, have been shown to produce ZnNPs ranging from 35 to 200 nm [32]. Additionally, *Fomes fomentarius* extracts containing flavonoids and alkaloids have yielded highly stable AgNPs (10–20 nm) with vigorous antioxidant activity [31].

### 3.4. Terpenoids and Other Metabolites

Terpenoids, abundant plant secondary metabolites, are mainly found in mushrooms and have gained significant attention for their role in the green synthesis of metallic nanoparticles, particularly AgNPs and AuNPs [37,38]. Ganoderic acids and other triterpenoids from *G. lucidum* contribute to NPs functionalization through their electron-rich chemical structure, which provides additional stability of NPs [29]. *G. lucidum* extract can produce stable silver NPs across a wide pH range, with the extract components potentially forming chemical bonds with the NP surface [34]. Furthermore, a broad spectrum of metabolites, including steroids, alkaloids, sesquiterpenoids, meroterpenoids, sterols, and vitamins, has been identified within mushroom extracts [39]. However, the exact functions of these compounds in NPs synthesis are not yet fully understood.

### 3.5. Unique Bioactives: Homogentisic Acid

A notable example involves homogentisic acid, a phenolic compound found in *Lactarius piperatus*, which has been directly linked to the synthesis of AgNPs with diameters ranging from approximately 33 to 64 nm [35]. Chromatographic analysis confirmed that this compound acts as a principal reducing agent, highlighting the potential of species-specific metabolites in controlling nanoparticle size and stability.

## 4. Antimicrobial Mechanisms of Myco-Nanoparticles

Myco-nanoparticles (myco-NPs), synthesized using bioactive compounds from edible and medicinal mushrooms, exhibit broad-spectrum antimicrobial activity. These antimicrobial effects are driven by three primary mechanisms: direct pathogen inhibition, indirect immune modulation, and synergistic enhancement with conventional antibiotics. These mechanisms are not mutually exclusive and may act in concert, depending on the nanoparticle composition and the microbial target. Myco-nanoparticles (myco-NPs) are prepared with bioactive molecules of edible and medicinal mushrooms, and they have broad-spectrum antimicrobial activity [40].

**Figure 2:**
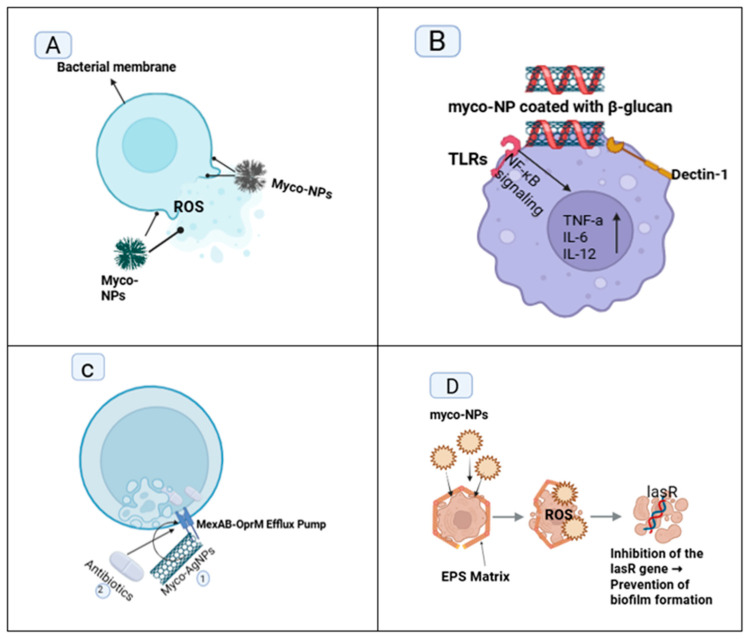
Antimicrobial Mechanisms of Myco-Nanoparticles: (**A**) Bacterial membrane rupture (Myco-NPs rupture bacterial membranes and generate ROS), (**B**) Immune cell activation (Myco-NPs interact with immune receptors (like TLRs and dectin-1) to activate macrophages and promote pro-inflammatory responses for pathogen clearance), (**C**) Synergy with antibiotics (Myco-NPs Enhance Antibiotic Efficacy by Inhibiting Efflux Pumps and Disrupting Membranes), (**D**) Biofilm penetration (Myco-NPs disrupt biofilms by targeting EPS, ROS production, and gene inhibition).

Four primary mechanisms govern their antimicrobial activities: direct inhibition of the pathogen, indirect immune modulation, enhancement of the action of conventional antibiotics through synergy, and disruption of biofilms, as illustrated in Figure 2 [15,41,42,43].

They are not independent mechanisms but work interactively, depending on the physicochemical properties of the particles, such as size, shape, charge, and surface chemistry, as well as the nature of the target microorganism [40]. Taken together, the intersection of the above biochemical and nano-structural properties makes myco-NPs a multifunctional and green platform to fight multidrug-resistant (MDR) microorganisms, which is a pressing global need today, given the emergence of increasing antimicrobial resistance (AMR) [41].

### 4.1. Direct Antimicrobial Effects

The inherent antimicrobial activity of myco-nanoparticles (myco-NPs) primarily occurs through mechanisms that disrupt bacterial cell integrity and hinder essential cellular functions [15]. Metallic nanoparticles synthesized using mushroom species, such as *Ganoderma lucidum*, *Pleurotus ostreatus*, and *Lentinus edodes*, exhibit intense bactericidal activities, predominantly through surface interactions with bacterial membranes [29,44,45].

Those nanoparticles, typically composed of silver (AgNPs), zinc oxide (ZnO NPs), or copper oxide (CuO NPs), bind to the surfaces of bacteria and disrupt the membrane structure, while causing increased permeability and subsequent leakage of intracellular components [15,45]. Such physical disruption is further reinforced through the minuscule nature and extensive surface area of the nanoparticles, which enable them to have intimate interactions with microbial membranes [46].

Beyond structural damage, myco-NPs induce biochemical stress within microbial cells by generating reactive oxygen species (ROS), including hydroxyl radicals (•OH), superoxide anions (O_2_^−^), and hydrogen peroxide (H_2_O_2_) [15]. These ROS attack cellular macromolecules, oxidizing membrane lipids, denaturing proteins, and fragmenting DNA, culminating in functional collapse and cell death [47].

In parallel, ROS interacts with intracellular proteins, leading to the oxidation of amino acid side chains, peptide backbone cleavage, and carbonylation. These oxidative modifications impair enzymatic activity and the stability of structural proteins, ultimately disrupting cellular metabolism and repair mechanisms [48].

Silver (AgNPs) and copper oxide nanoparticles (CuO NPs) are known to induce antimicrobial activity primarily through catalyzing ROS production via redox cycling at their surfaces, leading to oxidative stress within microbial cells. The generated ROS interacts with vital cellular components, causing widespread molecular damage. Proteins undergo oxidative modifications, such as carbonylation and backbone cleavage, which impair their enzymatic and structural roles [49].

Lipid peroxidation compromises membrane integrity, resulting in increased cell permeability. DNA is also a critical target, with ROS inducing strand breaks and base oxidation, ultimately inhibiting replication and transcription. This cumulative oxidative damage overwhelms microbial defense mechanisms, leading to cell death. Empirical data validate these mechanisms. AgNPs of *G. lucidum* with average diameters of ~15 nm exhibited intense bactericidal activity toward *Staphylococcus aureus* through membrane destruction as well as ROS-driven cytotoxic activity. These activities highlight the multifaceted nature of myco-NP antimicrobial activity and their potential as single-agent or adjuvant therapies for treating pathogenic bacteria [50]. Similar antibacterial effects of myco-synthesized nanoparticles have also been observed against other bacterial species, including *E. coli*, *Ps. aeruginosa*, *B. subtilis*, and *Enterococcus faecalis* [51,52], demonstrating that their antimicrobial activity extends beyond *S. aureus* and covers both Gram-positive and Gram-negative pathogens.

The antimicrobial efficacy of mushroom-derived nanoparticles (myco-NPs) often exhibits selective activity against specific bacterial taxa, primarily due to variations in cell wall architecture and surface charge. Gram-negative bacteria, such as *E. coli* and *Ps. aeruginosa*, possess an outer membrane rich in lipopolysaccharides, which can act as a partial barrier to nanoparticle penetration. In contrast, Gram-positive bacteria, including *S. aureus* and *B. subtilis*, have a thicker peptidoglycan layer with higher anionic charge density, promoting electrostatic attraction with positively charged myco-NPs and enhancing their susceptibility [53,54].

#### Differential Susceptibility of Gram-Positive and Gram-Negative Bacteria to Myco-NPs

The antimicrobial potency of mushroom-derived nanoparticles (myco-NPs) varies considerably between Gram-positive and Gram-negative bacterial strains, primarily due to differences in their cell wall architecture, surface charge, and permeability. Gram-positive bacteria such as *S. aureus* and *B. subtilis* possess a thick peptidoglycan layer (20–80 nm) that is rich in teichoic and lipoteichoic acids, conferring a net negative charge. This promotes stronger electrostatic attraction with positively charged nanoparticles (e.g., AgNPs, CuNPs), facilitating close surface contact and membrane disruption. In contrast, Gram-negative bacteria like *E. coli* and *Ps. aeruginosa* have a thinner peptidoglycan layer (7–8 nm) shielded by an outer membrane composed of lipopolysaccharides (LPS), which acts as a selective barrier to nanoparticle penetration [20,44,51].

Additionally, the composition of the outer membrane in Gram-negative species restricts diffusion of metal ions and reactive oxygen species (ROS), often resulting in lower susceptibility. However, smaller nanoparticles (<20 nm) and those with higher surface reactivity can penetrate these barriers more effectively, generating ROS within the periplasmic space and inducing oxidative stress [55].

### 4.2. Indirect Immunomodulatory Effects

Myco-nanoparticles (myco-NPs) produced from bioactive molecules obtained from mushrooms, specifically β-glucans and phenolic acids, have demonstrated potential immunomodulatory activity in addition to their direct antimicrobial activity [27]. Natural polysaccharides have been shown to interact through their pattern recognition receptors (PRRs), specifically Dectin-1 and Toll-like receptors (TLRs), which are expressed on innate immune cells, including macrophages and dendritic cells. Upon interaction, the immune signaling cascades are activated, which enhance the body’s defense mechanisms [27,56].

For instance, β-glucans from *Pleurotus* and *Lentinula* mushrooms have been shown to activate macrophages and promote their polarization to the pro-inflammatory M1 phenotype [57]. This phenotype has been associated with heightened phagocytic activity, increased cytokine production, such as TNF-α, IL-12, and IL-6, as well as iNOS upregulation, all of which collectively contribute to the efficient elimination of the pathogen. Similarly, iron oxide nanoparticles coated with β-glucan were found to have selective uptake by macrophages while simultaneously activating TLR-mediated NF-κB signaling cascades, which induce an inflammatory microenvironment conducive to the clearance of microorganisms [58].

Experimental studies in murine models further support these findings. Macrophages exposed to β-glucan-functionalized nanoparticles exhibited enhanced bacterial clearance, increased phagocytic efficiency, and modulation of cytokine profiles that favor antimicrobial responses [59].

### 4.3. Synergistic Enhancement with Conventional Antibiotics

Mushroom-based nanoparticles (myco-NPs) have emerged as potential adjuvants to traditional antibiotics, both by delivering direct antimicrobial activity and by regulating key mechanisms of resistance [60]. In particular, myco-NPs can inhibit bacterial efflux pumps and disrupt membrane integrity, thereby increasing the uptake and intracellular concentration of the drug [61].

Additionally, myco-synthesized silver nanoparticles (AgNPs), specifically those derived from fungi such as *L. edodes*, have demonstrated considerable synergistic effects when co-administered with antibiotics, including ciprofloxacin. No direct chemical conjugation occurs, yet the process of functional enhancement, where the membrane integrity of bacteria and efflux pumping systems, most notably the MexAB-OprM pump are interfered with by AgNPs, leading to improved ciprofloxacin intracellular penetration, does take place. Some recent experimental studies have revealed a 4–16 times reduced minimum inhibitory concentration (MIC) of ciprofloxacin against *Pseudomonas aeruginosa,* thereby exposing the potentiating capacity of AgNPs to overcome the barrier of antibiotic resistance [62].

### 4.4. Biofilm Disruption

Biofilms pose a substantial challenge in antimicrobial therapy due to their highly organized extracellular polymeric substance (EPS) matrix, which serves as a physical and biochemical barrier against antibiotic penetration. Mushroom-derived nanoparticles (myco-NPs) have shown considerable potential in disrupting these protective structures due to their unique physicochemical characteristics and surface functionalities [63].

One of the primary mechanisms by which myco-NPs exhibit antibiofilm activity is through the production of reactive oxygen species (ROS). ROS can oxidatively break down components of the EPS, including extracellular DNA (eDNA), proteins, and polysaccharides, thereby disrupting the structural integrity of the biofilm. Additionally, the tiny size and strong surface reactivity of myco-NPs enable them to penetrate deeper into biofilm layers, facilitating the detachment of immersed microbial cells and increasing the accessibility of the antimicrobial agent [64].

Analogously, fungal-derived nanoparticles have been shown to disrupt quorum-sensing mechanisms, which are central to biofilm maturation, by downregulating regulatory genes such as lasR and repressing autoinducer-mediated signaling [63].

These results indicate that myco-NPs are not acting as simple passive antimicrobial agents; instead, they actively break down complex microbial communities through multifactorial activities, providing a potential platform for biofilm-oriented treatments in both clinical and industrial applications [65].

Bioactive agents and nanoparticles (NPs) exert antimicrobial effects through various mechanisms, including indirect and synergistic modes of action. Indirectly, compounds such as β-glucans from *Pleurotus* and *Lentinula* species, along with iron oxide nanoparticles coated with polysaccharides, activate the host immune response by promoting macrophage M1 polarization and increasing cytokine levels, including TNF-α and IL-6, thereby enhancing immune clearance in murine models [66].

Synergistically, silver nanoparticles (AgNPs) derived from *L. edodes* combined with ciprofloxacin inhibit bacterial efflux pumps, increase drug uptake, and destabilize cell membranes, leading to a significant reduction in the minimum inhibitory concentration (MIC) of antibiotics against pathogens such as *Ps. aeruginosa* and *E. coli* [62].

In addition to their intrinsic antimicrobial activity, myco-NPs can be further enhanced through integration with herbal bioactives, a rapidly emerging field that merges nanotechnology with traditional medicine for synergistic therapeutic outcomes [67]. For instance, myco-nanoparticles, like zinc oxide or silver ones made using fungi such as *G. lucidum* or *Trichoderma harzianum* become even more powerful when paired with natural compounds like neem extract or curcumin [68].

## 5. Green Synthesis of Myco-Nanomaterials

### 5.1. Optimization of Synthesis Parameters

The green synthesis of nanoparticles (NPs) using mushroom extracts is a highly tunable process influenced by several key parameters, including pH, temperature, extract concentration, and metal salt concentration [69].

These factors play a pivotal role in controlling the morphology, size, stability, and bioactivity of the resulting nanoparticles. By modulating these parameters, researchers can optimize reduction kinetics, nucleation rates, and capping efficiency, all of which ultimately determine the functional properties of the synthesized nanomaterials, such as antimicrobial activity, biocompatibility, and colloidal stability [70]. In addition to synthesis conditions, the culture conditions of mushrooms significantly impact nanoparticle production.

**Figure 3:**
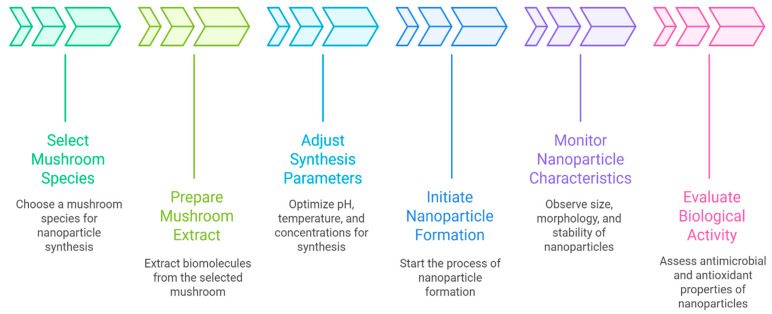
List of steps of using mushroom extract in preparing the nanoparticles.

Factors such as the species or strain used, cultivation method, developmental stage, and environmental conditions (e.g., substrate, light, and temperature) influence the biochemical profile of the mushroom extract, and consequently, the size, shape, and stability of the nanoparticles produced (Figure 3).

### 5.2. Effect of Mushroom Species

Among the most critical parameters is the choice of mushroom species, as each exhibits distinct capabilities in nanoparticle biosynthesis due to variations in their enzymatic and phytochemical content. Species from the genera *Pleurotus*, *Ganoderma*, and *Agaricus* have been widely studied for their ability to act as natural biofactories, reducing metal ions into stable nanoparticles through eco-friendly mechanisms [40,71,72]. Studies have demonstrated that species-specific effects can be addressed as follows:

- *Pleurotus* spp. (Oyster mushrooms) have been reported to produce silver nanoparticles (Ag-NPs) ranging from 2 to 100 nm, showing potent antimicrobial properties with applications in biomedicine and environmental remediation [73].
- *Ganoderma* spp. are noted for their high nanoparticle yield and safety profile, making them suitable for synthesizing various types of metal nanoparticles [71].
- *Agaricus* spp., widely appreciated for their nutritional and therapeutic properties, have shown potential in producing biologically active nanoparticles with enhanced antioxidant and antimicrobial activities [72].
- The biosynthesis mechanisms largely depend on the enzymes and phytochemicals secreted by mushrooms, which act as both reducing and stabilizing agents. Compounds such as flavones, phenolics, polysaccharides, and reductases contribute significantly to the reduction in metal ions and stabilization of the nanoparticles during formation [40,72].
- Further emphasizing the influence of species and cultivation is necessary, as both the mushroom strain and the method of cultivation play critical roles in determining nanoparticle characteristics. In their work, four species (*Chlorophyllum agaricoides*, *Coriolopsis trogii*, *Ganoderma* sp., and *Lentinus tigrinus*) were cultivated on potato dextrose agar (PDA) and used for AgNP synthesis. The resulting nanoparticles showed varying crystallite sizes (25.31 to 31.42 nm), reflecting the impact of species-specific biochemical profiles. The use of cultivated mushrooms under controlled conditions ensured reproducibility and consistency in nanoparticle production. Analytical characterization confirmed that bioactive molecules in the extracts were key to both the reduction and stabilization processes, and also contributed to the antimicrobial and antioxidant activities observed [74].
- Similarly, the use of *A. bisporus* as a biological reducing agent in the synthesis of silver nanoparticles. The study emphasized that the specific strain composition, rich in proteins, polysaccharides, and phenolics, played a vital role in both reducing Ag^+^ to Ag^0^ and stabilizing the nanoparticles. The authors highlighted that variations in the type and concentration of these biomolecules among mushroom strains directly affect the efficiency, size, and morphology of the resulting nanoparticles [18].

### 5.3. Effect of Mushroom Extract Concentration

The concentration of mushroom extracts plays a critical role in determining the size, morphology, distribution, and stability of biosynthesized nanoparticles. As the concentration of the extract increases, the availability of reducing and stabilizing biomolecules, such as proteins, polysaccharides, and phenolic compounds, also increases, influencing the nucleation and growth of nanoparticles. Several studies have demonstrated that higher concentrations of mushroom extract can lead to smaller and more uniformly distributed nanoparticles. For example, *Psathyrella candolleana* extract, when used at increasing concentrations, reduced the crystallite size of ZnO-NPs from 51 nm to 19 nm [75]. Similarly, the use of *Termitomyces heimii* extract resulted in CdS-NPs with diameters below 5 nm, confirming the impact of extract concentration on particle size [76]. In the case of silver nanoparticles (Ag-NPs), *A. bisporus* extracts produced particles ranging from 8 to 50 nm, with size variations directly influenced by extract concentration [77]. Higher concentrations of mushroom extracts not only enhance the reduction in metal ions but also improve the biological activity and colloidal stability of the synthesized nanoparticles [78].

Moreover, increased extract concentration has been linked to a more consistent particle size distribution, as evidenced by transmission electron microscopy (TEM) images showing greater uniformity [70,71]. Optical properties, such as bandgap energy, have also been reported to shift in response to concentration changes, reflecting variations in nanoparticle morphology and quantum confinement effects [75].

However, excessively high concentrations of extracts can have undesirable effects, such as nanoparticle agglomeration and reduced synthesis efficiency. This was tested before at different extract-to-AgNO_3_ ratios (0.5:1, 1:10, and 1.5:10) for the synthesis of Ag-NPs. They found that the 1:10 ratio yielded optimal results, inducing color change within 60 min and avoiding precipitation. In contrast, the 1.5:10 ratio led to visible precipitate formation, indicating instability at higher extract concentrations. Interestingly, their results also suggested that lower concentrations of both extract and silver precursor favored the formation of smaller nanoparticles [79]. Contrary to the general trend of smaller nanoparticles at higher extract concentrations, increasing the amount of mushroom extract was observed to lead to larger particle sizes, likely due to reduced availability of silver ions in the reaction mixture. This highlights the importance of balancing extract and metal ion concentrations to optimize synthesis outcomes [80].

**Figure 4:**
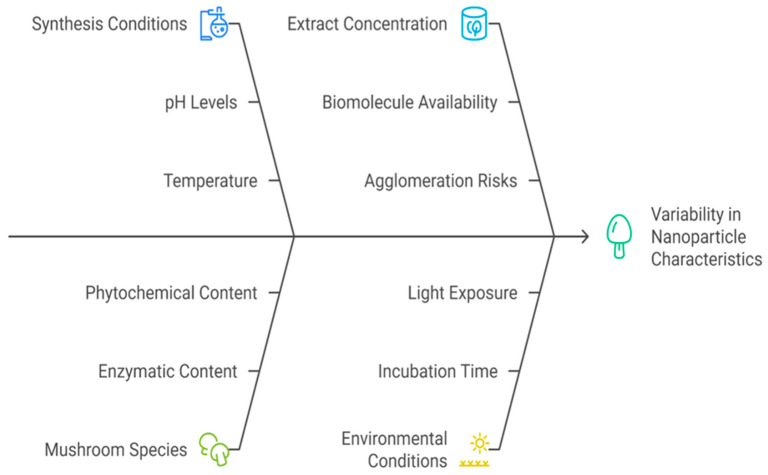
Summary including the main factors controlling the mushroom synthesis of nanoparticles.

In summary, the concentration of mushroom extract is a critical parameter that requires careful optimization. While moderate increases in concentration typically improve nanoparticle size control and stability, excessive amounts may lead to aggregation or undesired morphological changes. Achieving the ideal ratio between extract and metal precursor is essential for tailoring nanoparticle characteristics for specific applications (Figure 4).

### 5.4. Environmental Conditions

The biosynthesis of NPs using mushroom extracts is highly sensitive to environmental factors such as pH, temperature, incubation time, agitation, and external energy sources. These parameters influence not only the physicochemical characteristics of the nanoparticles—such as size, morphology, and stability, but also their biological functionality. Optimizing these conditions is essential to ensure efficient metal ion reduction, uniform particle formation, and enhanced biological activity. The environmental conditions can be highlighted as follows:

The pH of the reaction medium plays a pivotal role in nanoparticle synthesis by affecting enzyme activity, the ionization of biomolecules, and the system’s reduction potential. For gold nanoparticles (Au-NPs), studies have shown that alkaline conditions (pH 10–11) yield ultrasmall and uniformly distributed particles, approximately 2.6 ± 1.1 nm in size [81]. Similarly, the synthesis of silver nanoparticles (Ag-NPs) from *A. bisporus* was optimized under higher pH conditions, which favored the formation of smaller nanoparticles [77].

The pH range from 4 to 11 significantly influenced nanoparticle size, with alkaline conditions promoting rapid synthesis and greater uniformity. However, excessively high pH values may lead to instability and aggregation, emphasizing the need for controlled optimization based on the specific nanoparticle and mushroom species involved [82].

Temperature is another key factor influencing nanoparticle synthesis. Elevated temperatures enhance the kinetic energy of reacting molecules, accelerating the reduction of metal ions and promoting the formation of smaller, monodisperse nanoparticles. For instance, AgNPs synthesized at 90 °C under basic pH conditions using fungal cell-free extracts were reported to be in the range of 3 to 17 nm [83]. In the case of zinc oxide nanoparticles (ZnO NPs), isotropic particles (~25 nm) were produced at temperatures above 200 °C, while lower temperatures led to larger and more anisotropic particles [84]. This indicates that higher temperatures favor the synthesis of smaller, more uniform ZnO NPs. The temperature, along with extract concentration, significantly influences the crystallite size and morphology of ZnO nanoparticles [75].

Energy input in the form of microwave irradiation has also been shown to affect nanoparticle synthesis. *A. bisporus* extract was used to synthesize Au-NPs, and it was found that microwave exposure time and HAuCl_4_ concentration significantly influenced particle size, concentration, and polydispersity index (PDI). This suggests that controlled microwave irradiation can be a useful tool for fine-tuning nanoparticle properties during green synthesis [22].

Agitation during synthesis facilitates homogeneous distribution of biomolecules, improves mass transfer, and enhances the reaction kinetics between the mushroom extract and the metal precursor. Shaking the reaction mixture at 150 rpm during overnight incubation improved the uniformity and size distribution of Ag-NPs synthesized from *A. bisporus* [18]. This mechanical mixing helps avoid localized concentration gradients and ensures efficient interaction between reducing agents and metal ions, leading to more consistent nanoparticle synthesis [78].

Incubation time is another critical variable, as it determines the extent of metal ion reduction. Overnight incubation at room temperature in the dark ensured the complete formation of Ag-NPs. Darkness was crucial to prevent photo-degradation or unwanted photoreactions of bioactive compounds. Room-temperature conditions also reflect the eco-friendly, mild nature of the biosynthesis process [18].

Impurities, often originating from secondary metabolites in mushroom extracts, can also influence nanoparticle synthesis. These co-extracted substances may interfere with the uniformity and stability of the nanoparticles. Impurities can cause aggregation during sample preparation, particularly during solvent evaporation, thereby altering the nanoparticle size and morphology. X-ray diffraction (XRD) analysis confirmed the presence of these impurities, underscoring their potential to negatively affect the quality and reproducibility of nanoparticle synthesis [74]. The summarized optimal synthesis parameters for different mushroom species and metal precursors, including their characteristic nanoparticle properties, are presented in Table 2.

### 5.5. Advantages of Myco-Synthesis over Conventional Methods

Myco-synthesis presents several advantages over traditional chemical and physical methods for nanoparticle production. Green synthesis of nanoparticles by mushrooms has several benefits, including low-cost production, energy efficiency, protecting human health and communities, safe products, fewer accidents, economical and competitive advantages, and less waste; therefore, it is also called eco-friendly and used in the pharmaceutical industry and other biomedical applications (Table 3). The comparison between myco-synthesis and other traditional methods for producing various nanoparticles has been reported by numerous researchers, including Ag-NPs [88], magnetic Fe_3_O_4_-NPs [89], and CaO-NPs [69].

## 6. Characterization of Myco-Nanomaterials

The effectiveness of nanoparticles (NPs) depends on complex structure property relationships influenced by synthesis conditions and mushroom phytochemicals [13]. To understand these links, a thorough physicochemical analysis is essential. Advanced analytical methods confirm NPs formation and offer vital information on size, shape, crystallinity, surface chemistry, and colloidal stability, all of which are crucial for their biological functions, especially antimicrobial activity [98].

### 6.1. Dynamic Light Scattering (DLS): Hydrodynamic Size and Surface Charge

DLS is a fundamental method for measuring the hydrodynamic diameter and zeta potential of myco-NPs in water-based solutions. Unlike the core size determined by electron microscopy, the hydrodynamic size encompasses the solvation layer and bio-organic corona, providing a more accurate measure of the particles’ adequate size in biological environments [99]. Nanoparticles ranging from 10 to 100 nm tend to have better cellular interactions, promoting membrane penetration and antimicrobial action [100]. Zeta potential values (generally above ±30 mV) are linked to colloidal stability and influence electrostatic interactions with negatively charged microbial surfaces, affecting adhesion, uptake, and bactericidal effectiveness [101].

### 6.2. Transmission and Scanning Electron Microscopy (TEM/SEM): Morphology and Core Size

TEM and SEM are still the gold standards for visualizing nanoparticle morphology and primary size distribution. TEM provides high-resolution images of internal nanostructures, lattice fringes, and crystallinity, while SEM is better suited for topographical and surface architecture analysis [102]. Morphological features, such as sphericity, aspect ratio, and aggregation, influence antimicrobial activity by modifying the contact surface area, cellular internalization, and mode of action, whether through physical puncturing or endocytosis [103]. Spherical AgNPs with a narrow size distribution often exhibit higher activity against both Gram-positive and Gram-negative bacteria due to optimized surface interactions.

Beyond spherical AgNPs, myco-synthesized nanoparticles exhibit diverse morphologies and compositions. TEM and SEM analyses have revealed hexagonal and rod-shaped ZnO NPs from *L. edodes* and *Psathyrella candolleana*, cuboidal CuO NPs from *Aspergillus* species, and irregular FeNPs from *Pleurotus florida* [104]. These morphological variations influence surface area, reactivity, and antimicrobial efficiency, with smaller or anisotropic shapes often showing enhanced membrane interaction and ROS generation [103].

### 6.3. X-Ray Diffraction (XRD): Crystallinity and Phase Identification

XRD offers crucial insights into the crystal structure, phase purity, and crystallite size of myco-NPs. Sharp diffraction peaks signal high crystallinity, which is often linked to improved redox properties and catalytic functions. A smaller crystallite size increases the surface area and number of active sites, enhancing electron transfer between the nanoparticle and microbial membrane components, which in turn accelerates reactive oxygen species (ROS) generation and boosts antimicrobial effectiveness [105,106]. The Debye–Scherrer equation enables the calculation of average crystallite sizes, which are related to electron transfer, a process essential for antimicrobial actions such as reactive oxygen species (ROS) production [107].

The absence of secondary phases or impurities demonstrates the effectiveness of mushroom phytochemicals in producing pure and uniform nanostructures [108].

### 6.4. Fourier-Transform Infrared Spectroscopy (FTIR): Surface Functionalization and Capping Agents

FTIR analysis indicates the types of biomolecules that cap and stabilize nanoparticles. These bio-reductants, such as mushroom polysaccharides, polyphenols, proteins, flavonoids, and terpenoids, play a crucial role in controlling nanoparticle nucleation, growth, and dispersion [79]. Commonly observed functional groups include hydroxyl (–OH), carbonyl (C=O), carboxyl (–COOH), and amine (–NH_2_), which suggest hydrogen bonding and electrostatic stabilization [109]. These surface groups work in conjunction with metal cores to enhance antimicrobial activity by inactivating enzymes, disrupting membranes, and breaking down biofilms [110].

### 6.5. UV-Visible Spectroscopy: Surface Plasmon Resonance (SPR) and Optical Monitoring

UV-Vis spectroscopy is a quick and non-destructive method for monitoring NPs synthesis by detecting SPR bands [111]. For example, AgNPs generally show clear absorption peaks in the 400–450 nm range [112], while AuNPs have SPR around 520–540 nm. The position, strength, and width of these peaks provide indirect insights into particle size, distribution, and clustering [113]. Shifts in the SPR band over time can also reveal consistency in synthesis and long-term colloidal stability, which is a key factor for both clinical and environmental uses [114]. A comparative summary of mushroom species, NPs used, and antimicrobial effects is presented in Table 4, highlighting the diversity and potential of myco-NPs as precision-engineered antimicrobial agents.

**Figure 5:**
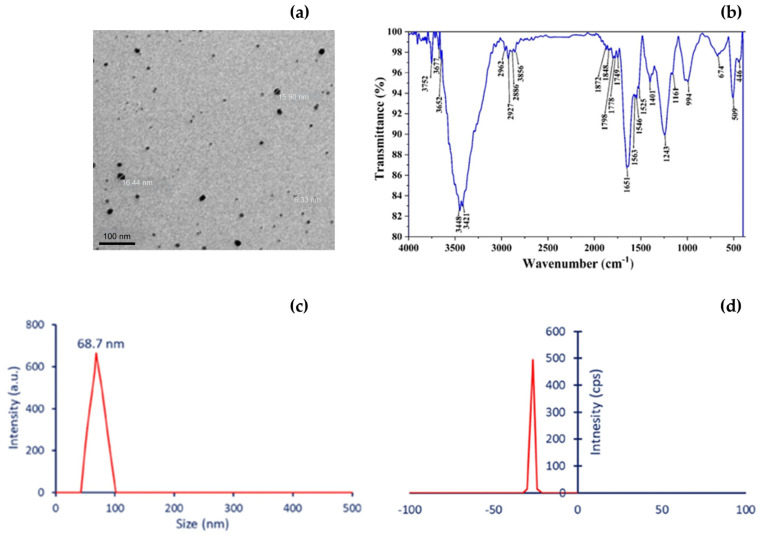
Representative characterization of biosynthesized myco-nanoparticles. (**a**) TEM image showing spherical AgNPs from *A. flavipes* AUMC 15772 with size distribution ranging from 8–26 nm with 100 nm scale [121], (**b**) FTIR spectrum of AgNPs [122], (**c**) DLS of CuO-NPs from *A. terreus* 68.7 nm [118], and (**d**) Zeta potential measurement of CuO-NPs from *A. terreus*, revealing a sharp peak around –32 mV [118].

The synthesized myco-NPs were characterized using TEM, FTIR, DLS, and zeta potential analysis, confirming their structural morphology, surface functionalization, and colloidal stability (Figure 5).

Significant gaps remain, particularly the lack of pharmacokinetic and pharmacodynamic data to clarify their distribution and metabolism, along with the absence of well-defined regulatory frameworks for naturally synthesized nanomaterials [123,124].

## 7. Safety and Regulatory Considerations

Mushroom-derived nanomaterials hold immense promise; however, their clinical success hinges on robust safety evaluation and the development of appropriate regulatory frameworks. Bridging the gap between biological origin and nanoscale engineering requires interdisciplinary collaboration across toxicology, nanotechnology, pharmacology, and regulatory science. Although myco-NPs are considered biocompatible, their safety depends on several factors, including particle size, surface charge, mushroom species, extract composition, and their accumulation and clearance profiles. The accumulation of NPs can damage body cells, although some studies have shown no life-threatening toxicity [125].

Studies have shown that myco-NPs often exhibit lower toxicity and higher biocompatibility than conventionally synthesized metallic NPs with better stability, longer shelf life, and greater biological activity [40]. For example, *A. bisporus* mushroom extract-based Ag NPs have demonstrated vigorous antibacterial activity. Moreover, they were noncytotoxic to normal human dermal fibroblast cells [126]. In contrast, carbon quantum dots derived from *Suillus luteus* exhibited partial cytotoxicity in human epithelial cells, whereas *P. ostreatus* and *A. bisporus* did not [127]. The pharmacokinetics and potential toxicity of these NPs are influenced by many variables, including dosage, route of exposure (topical, intramuscular, intradermal, parenteral, and subcutaneous), and physicochemical characteristics (morphology, composition, size, charge, etc. [66].

Metal-based nanoparticles can accumulate in the body and harm cells by inducing oxidative stress, disrupting membranes, and impairing mitochondrial function; however, studies have shown that biosynthesized AgNPs, ZnO NPs, and CuO NPs generally exhibit low cytotoxicity and pose no life-threatening risks when used at controlled doses [128].

Despite these promising findings, further research is still needed to fully benefit from myco-NPs, as their side effects remain poorly understood, which limits their clinical application. The regular use of myco-NPs to treat diseases caused by bacteria resistant to many drugs will be made possible by unraveling their toxicity through thorough in vivo and clinical trials [129]. Furthermore, major gaps persist, such as the limited pharmacokinetic and pharmacodynamic data needed to understand their distribution and metabolism, along with the absence of clearly established regulatory pathways for naturally synthesized nanomaterials [123].

During the clinical translation and commercialization process, nanotechnology and nanomaterials used in healthcare must be regulated as either nanomedicines or nanotechnology-based medical devices. Nanomedicines must undergo a thorough approval process before they can be used in clinical settings. Preclinical research and clinical trials are employed in this procedure to ensure the quality, safety, and effectiveness of the products. This procedure is governed by regulatory agencies such as the FDA, the European Medicines Agency (EMA), and China’s National Medical Products Administration (NMPA), which require rigorous testing and review to meet their criteria [130]. However, the rise of myco-NPs involves the development of regulatory frameworks relevant to nanotechnology, taking into account their special characteristics and uses. In this situation, applying OECD guidelines for nanomaterial toxicity testing (e.g., inhalation, dermal, oral exposure) becomes essential to ensure a standardized assessment of their safety profile [131].

**Figure 6:**
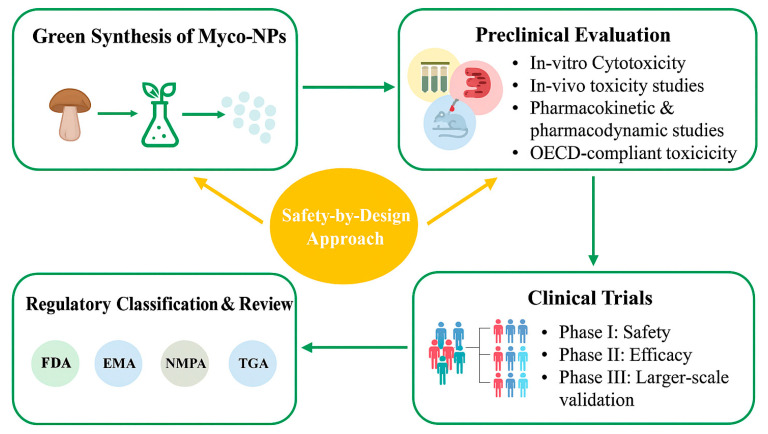
Safety and Regulatory Workflow for Myco-NPs.

Additionally, a “safety-by-design” strategy should be employed in the future development of myco-NPs to ensure safe clinical translation, as summarized in Figure 6. This approach aims to mitigate the unwanted effects of NPs by incorporating knowledge of their adverse impact on the environment and human health into the design process of the desired NPs [132].

Although very small metal nanoparticles can induce mutagenicity at high, uncontrolled doses, green-synthesized myco-NPs use natural capping agents and optimized concentrations that limit DNA interaction and oxidative stress, enabling safe antimicrobial application [133].

## 8. Challenges and Future Perspectives

The exploration of green-synthesized nanomaterials derived from edible and medicinal mushrooms presents a promising strategy to tackle the mounting challenge of antimicrobial resistance (AMR), a significant and evolving threat to global public health. While current literature emphasizes the potential of these materials in biomedicine and food safety, several critical challenges must be addressed to realize their full potential and guide future developments in this field.

One of the foremost challenges is the systematic evaluation of the safety and efficacy of green-synthesized nanoparticles. Although nanoparticles synthesized using plant-based methods exhibit significant pharmacological properties such as anti-acne and anti-cancer activities, comprehensive safety assessments remain insufficient. Future perspectives must emphasize the development of standardized testing protocols to evaluate antioxidant, antibacterial, and cytotoxic effects to ensure these materials can be safely integrated into clinical and commercial applications [134].

Building on these concerns, there is a necessity for in-depth biological screening and precise characterization of nanoparticles, particularly silver and gold nanoparticles. The current lack of consistency in nanoparticle characterization poses a barrier to their reliable application in nanomedicine. Future research should prioritize the establishment of harmonized characterization techniques and toxicological assessments across a range of biological systems to guarantee reproducibility and biocompatibility [135].

Another critical issue: the variability and lack of standardization in the green synthesis of zinc oxide nanoparticles (ZnO-NPs). Although ZnO-NPs show promise in applications such as antimicrobial bandages and drug delivery systems, inconsistent production methods can result in significant variability in particle size, morphology, and efficacy.

Addressing these inconsistencies through the development of scalable, reproducible synthesis protocols is vital for the translation of laboratory findings into real-world applications [136].

The integration of advanced biotechnological tools is identified as a future direction in which omics-based approaches are being applied to enhance the yield and efficacy of bioactive compounds in mushrooms. Their findings suggest that incorporating systems biology into nanoparticle research can lead to more efficient discovery and production of valuable metabolites, paving the way for more targeted pharmaceutical interventions. However, the challenge lies in bridging the gap between laboratory-scale research and industrial-scale applications, which requires robust bioprocess optimization [137].

The practical applications of mushroom-derived nanoparticles in the realm of food safety, particularly using *A. bisporus*. Their study illustrates the effectiveness of these nanoparticles in combating foodborne pathogens, yet also highlights significant hurdles such as the optimization of synthesis methods and the elucidation of action mechanisms. Future efforts must focus on enhancing biocompatibility, ensuring consumer safety, and developing efficient production models for commercial scalability [18].

Collectively, these studies outline a trajectory marked by both promise and complexity. Key challenges moving forward include the need for standardization in synthesis and characterization, comprehensive safety evaluations, and the development of scalable production strategies. Addressing these issues will be crucial for translating the potential of green-synthesized nanomaterials from medicinal mushrooms into impactful solutions for AMR and beyond. As research advances, interdisciplinary collaboration and integration of emerging technologies will be vital in overcoming current limitations and unlocking new applications in nanomedicine and food biotechnology.

Green-synthesized myco-NPs are more environmentally safe than conventional metal nanoparticles, as their natural capping agents enhance biodegradability and reduce ecological persistence and bioaccumulation [44].

## 9. Conclusions

Mushroom-derived nanoparticles, known as myco-NPs, are gaining attention as an environmentally friendly and innovative approach to addressing the growing threat of antimicrobial resistance. These nanoparticles are formed using natural compounds from fungi, which not only help stabilize and reduce the particles during synthesis but also contribute to their antimicrobial and immune-enhancing effects. Their actions include damaging microbial cell membranes, generating reactive oxygen species, modulating immune responses, boosting the effects of antibiotics, and penetrating biofilms. Their green synthesis process supports the goals of sustainable chemistry and the circular bioeconomy. However, despite promising laboratory findings, the clinical application of myco-NPs faces several hurdles. Most current studies are limited to in vitro experiments, which do not provide a complete understanding of how these nanoparticles behave in living organisms, including their distribution, metabolism, and long-term safety.

Further challenges include inconsistencies between production batches, unclear toxicity limits, and the absence of standardized regulatory frameworks. Future research should focus on improving production consistency, lowering toxicity through biocompatible surface modifications, and developing universal testing methods to evaluate safety and effectiveness. It is also essential to assess the environmental impact and sustainability of large-scale production. Progress in this field will depend on strong collaboration among experts in microbiology, mycology, nanotechnology, clinical medicine, and regulatory science to bring mushroom-based nanotechnology from the lab to real-world use as a safe and effective antimicrobial solution.

## Figures and Tables

**Table 1:** Bioactive compounds in mushrooms for the synthesis of nanoparticles (NPs).

Compound Class	Mushroom Source(s)	Key Molecules	Role in NP Synthesis	Nanoparticle Size (nm)	References
Polysaccharides	*A. bisporus*, *Phellinus linteus*, *P. ostreatus*, *G. lucidum*	β-glucans, chitin, mannogalactans	Reduction and capping agents; stabilization	10–70 (AgNPs)	[27,28,29]
Proteins & Peptides	*G. lucidum*, *Volvariella volvacea*	Proteins (amide linkages, amino acids)	Reduction via amine and carboxylate groups; surface capping	2–70 (AgNPs, AuNPs)	[24,25,30]
Phenolics & Flavonoids	*L. edodes*, *Fomes fomentarius*, *Schizophyllum commune*	Catechins, quercetin, phenolic acids	Antioxidant reduction in metal ions; surface stabilization	10–20 (AgNPs), 35–200 (ZnO NPs)	[31,32,33]
Terpenoids	*G. lucidum*	Ganoderic acids, triterpenoids, steroids	Electron donation for reduction; surface functionalization	Not specified	[29,34]
Unique Phenolics	*Lactarius piperatus*	Homogentisic acid	Principal phenolic reducing agent; NP stabilization	33–64 (AgNPs)	[35]
Mixed Compounds	*G. lucidum*	Steroids, alkaloids, sesquiterpenoids, vitamins	Synergistic contribution to reduction and capping	Not specified	[29]

**Table 2:** Summarizes the main features of using different species of mushrooms in nanoparticle production and the optimal conditions for such synthesis.

Nanoparticle	Mushroom Species	Extract Concentration	Key NP Properties	Ref.
AgNPs	*Pleurotus* spp.	Not specified	2–100 nm; spherical; antimicrobial, anticancer, dye degradation	[73]
FeNPs	*Pleurotus florida*	Not specified	Color change; antimicrobial vs. bacteria & fungi; comparable to antibiotics	[85]
Metal NPs	*Pleurotus* spp.	Not specified	Intra-/extracellular routes; eco-friendly; antimicrobial, anticancer, antioxidant	[44]
ZnNPs	*Pleurotus sajor caju*	1 mM ZnSO_4_	SPR: 300 nm; spherical; 7–13 nm; FTIR: 675–3675 cm^−1^	[86]
ZnO NPs	*Daedalea* sp.	10 g extract + Zn acetate (1.834 g/100 mL)	14.6 nm; irregular, agglomerated; hexagonal (XRD); FTIR phenolics; biocompatible	[87]
ZnO NPs	*Psathyrella candolleana*	Varying concentrations	19–51 nm; sword-like (TEM); antibacterial; photocatalytic MB degradation (80%/60 min)	[75]

**Table 3:** Comparison: Myco-Synthesis vs. Conventional Synthesis of NPs.

Criteria	Myco-Synthesis of NPs	Conventional Methods of NPs	Refs.
Methodology	Uses fungi in the live cells or extracts as reducing or stabilizing agents	In chemical methods, it relies on reducing agents (e.g., NaBH_4_, citrate, vitamin C). In physical methods by laser ablation, ball milling, grinding, or evaporation–condensation	[89,90]
Reducing agents	By fungal exudated biomolecules such as amino acids, proteins, or enzymes (mainly reductases), metabolites, and polyphenolic as the reducing agents during the formation of NPs	Mainly in the chemical methods: harsh chemicals (e.g., hydrazine, sodium citrate, ascorbic acid)	[91,92]
Yield, stability & NPs size control	Moderate (depends on fungal strain, culture conditions), free from impurities with higher yields, eco-friendly, simple, and cost-effective	High (precise control via concentration/pH/temperature), contamination of the final NPs with chemicals could be observed, along with the production of hazardous by-products.	[92,93]
Solvents and stabilizing agents	Aqueous (water-based), natural fungal biomolecules (proteins, polysaccharides)	Often, organic solvents (e.g., toluene, hexane) are used. Synthetic surfactants/polymers (e.g., PVP, PEG)	[94]
Costs+ energy requirements	Low costs as they use biomass or waste substrates Low energy requirement as it uses the ambient temperature and pressure	High costs due to the expensive chemicals, energy-intensive, and others High energy requirements, such as high temperature, pressure, and vacuum, for physical methods	[70]
Environmental impacts	Safe product, competitive advantages, biodegradable byproducts, economical, eco-friendly as lesser waste	Toxic waste may contain heavy metals, solvents, and non-degradable materials, posing environmental pollution and adverse effects on ecosystems.	[70,91]
Scalability	Challenging for large-scale production	Well-established for industrial scale.	[70]
Applications	Biochemical, biomedicine, drug delivery, antimicrobials, eco-remediation, photodegradation ability, industrial biofortification,	Electronics, catalysis, optics, biomedical imaging and healthcare applications, energy storage applications,	[70,95,96,97]

**Table 4:** Characterization of Myco-Nanoparticles and Their Antimicrobial Properties.

Mushroom Species	NPs	Size (nm)	Shape	Effective Against	Ref.
*A. bisporus*	ZnO-NPs	15–25	Spherical/hexagonal	*B. cereus*, *E. coli*, *E. faecium*, *P. aeruginosa*, *A. niger*, *P. polonicum*, *P. ultimum*, *V. dahliae*	[115]
*A. carneus* MAK259	Ag-NPs	5–26	Spherical	*P. aeruginosa*	[116]
*A. fumigatus* SM4	CuO-NPs	20–150	Spherical	*S. aureus*, *B. subtilis*, *P. aeruginosa*, *E. coli*, *C. albicans*	[117]
*A. terreus*	Cu-NPs	4–45	Spherical	Klebsiella, *E. coli*	[118]
*Aspergillus* sp. JAWF3	CuO-NPs	35–130	Cuboid	*E. coli*, *P. aeruginosa*, *S. aureus*, *B. subtilis*	[119]
*F. fujikuroi* MED14	Se-NPs	10–19	Spherical	*E. coli*, *B. cereus*	[120]

## Data Availability

Not applicable.
